# Overcoming cisplatin resistance in osteosarcoma through the *miR-199a*-modulated inhibition of HIF-1α

**DOI:** 10.1042/BSR20170080

**Published:** 2019-11-15

**Authors:** Ajimu Keremu, Abudureyimu Aini, Yusufuaji Maimaitirexiati, Zhilin Liang, Pazila Aila, Paizila Xierela, Aikebaier Tusun, Hanikezi Moming, Aihemaitijiang Yusufu

**Affiliations:** 1 Department of Orthopedic, First People’s Hospital of Kashgar Area, No 120 Yingbin Road, Kashgar City, Xinjiang 844000, China; 2 Department of Micro Reconstruction Surgery, The First Affiliated Hospital of Xinjiang Medical University, No 137 Liyushan Road, Urumqi, Xinjiang 830054, China

**Keywords:** cisplatin sensitivity, HIF-1α, microRNA, miR-199a, osteosarcoma

## Abstract

Dysregulation of miRNAs has been shown to contribute to multiple tumorigenic processes, as well as to correlate with tumour progression and prognosis. *miR-199a* has been shown to be dysregulated in multiple tumour types. However, the association between *miR-199a* and the chemoresistance features of osteosarcoma are not well understood, the target genes for *miR-199a* and the regulatory mechanisms are also unknown. In the present study, we demonstrated that *miR-199a* is expressed at low levels in osteosarcoma cells and patient samples. By the selection and establishment of cisplatin resistant osteosarcoma cell line, we observed a correlation between *miR-199a* and cisplatin resistance in osteosarcoma cells: resistant cells exhibit attenuated *miR-199a* expressions and exogenous overexpression of *miR-199a* sensitizes osteosarcoma cells to cisplatin. Moreover, we identified *HIF-1α* as a direct target for *miR-199a*. Intriguingly, cisplatin resistant osteosarcoma cells display significantly elevated *HIF-1α* expression under hypoxia. We report here overexpression of *miR-199a* resensitizes cisplatin resistant cells to cisplatin through inhibition of *HIF-1α in vitro* and *in vivo*. Finally, by analysing the clinical osteosarcoma patient samples, we demonstrate a reverse correlation between *miR-199a* and *HIF-1α* mRNAs. Our study will provide mechanisms for the miRNA-mediated anticancer therapy and *miR-199a* may be considered a promising therapeutic agent for osteosarcoma patients who fail to respond to conventional chemotherapy.

## Introduction

miRNAs are a class of small non-coding, single-stranded endogenous RNA fragments that repress target protein translation by base pairing to the 3′-untranslated mRNA region [1,2]. miRNAs have emerged as important regulators involved in multiple cellular processes such as cell proliferation, apoptosis and autophagy [3,4]. The effects of miRNAs on tumorigenesis and cancer progression have been revealed that miRNA expressions are significantly up- or down-regulated in tumour tissues, resulting in a prominent diagnostic and prognostic tool [5,6]. Moreover, miRNAs have been reported to be involved in drug resistance. One study showed that *miR-125b* is correlated with Taxol-resistant breast cancer cells and inhibition of *miR-125b* could resensitize Taxol-resistant cells [7]. Another study illustrated that *miR-34a* overexpression results in attenuated chemoresistance to the camptothecin by targeting *SIRT1* gene [8]. Therefore, elucidating the critical functions of miRNAs in tumour progression is important to discover novel antitumour agents.

Osteosarcoma which arises from mesenchymal cells is the most common form of malignant bone tumour and occurs predominantly in adolescents and young adults [9]. Currently, a combination of therapies that include surgery, chemotherapy and radiation therapy is the most common therapeutic plan for osteosarcoma [10]. However, patients with recurrent or metastatic osteosarcomas still have very poor prognosis [11]. Among the antitumour agents, although cisplatin is one of the most effective drugs against osteosarcoma, outcomes remain poor for most patients who developed chemoresistance [12]. Thus, understanding the molecular mechanisms involved in the chemoresistance of osteosarcoma cells is a critical stage to improve the treatments.

*miR-199a* has been described to be deregulated after cisplatin treatment in cancer cell lines [13], suggesting that it may be a therapeutic target. However, the molecular mechanisms underlying this process are not well understood and the functions of *miR-199a* in osteosarcoma have not been clearly illustrated. In the present study, we focus on the roles of *miR-199a* in the cisplatin sensitivity in osteosarcoma. Moreover, the downstream target of *miR-199a* will be identified and we will investigate whether overexpression of *miR-199a* could sensitize cisplatin resistant osteosarcoma cells. We supposed that *miR-199a* could be a promising therapeutic agent in osteosarcoma.

## Materials and methods

A total of 20 osteosarcoma patient samples and their matched adjacent normal tissues were obtained from osteosarcoma patients at the Department of Orthopedic, First People’s Hospital in Kashgar area (Xinjiang, People’s Republic of China). The study was approved by the ethics committee of Department of Orthopedic, First People’s Hospital in Kashgar area (Xinjiang, People’s Republic of China). Written informed consents were obtained from all the patients. Tissue samples were collected at surgery, immediately frozen in liquid nitrogen and stored until analysis in the present study.

The human osteosarcoma cell lines (MG-63, U-2OS and SaoS-2) were purchased from the American Type Cell Culture Collection (Manassas, VA, U.S.A.). Human normal osteoblast cell line NHOST, hFOB and HOB were purchased from the Cell Bank of Chinese Academy of Sciences (Shanghai, China) and grown in Dulbecco’s Modified Eagle’s Medium (DMEM, Gibco, U.S.A.) supplemented with 10% FBS (Sigma, U.S.A.), 100 units/ml of penicillin streptomycin (Invitrogen, Carlsbad, CA). Cells were cultured in a humidified incubator at 37°C with 5% CO_2_ and 95% air. Hypoxia was performed in hypoxic chamber that was filled with gas mixture of 95% N_2_ and 5% CO_2_ for 24 h, then returned to regular 37°C, 5% CO_2_ and 95% air and regular cell culture medium.

### Antibodies and reagents

Rabbit monoclonal antibody anti-HIF-1α was purchased from Cell Signaling (#14179, Danvers, MA, U.S.A.); mouse monoclonal antibody anti-β-actin was purchased from Cell Signaling (#3700, Danvers, MA, U.S.A.). Cisplatin was purchased from Sigma–Aldrich (Shanghai, China) and stored as 20 mM solution in ddH_2_O at –20°C and diluted with DMEM medium prior to use.

### Selection of cisplatin resistant cell line

The selection of cisplatin resistant osteosarcoma cells was performed according to a recent report [14]. Briefly, SaoS-2 parental cells were treated with increased concentrations of cisplatin from 0.5–25 µM for the selection of survival cells. The treatments continued for 3 months and survival clones were pooled for the following assays in the present study.

### Real-time PCR

Total RNA was extracted from osteosarcoma cells by the TRIzol method as recently described [14]. The reverse transcription reaction was performed to reverse transcribe RNA into cDNA using an oligo (dT) primer from the miScript II RT Kit (Qiagen, Hilden, Germany). A total of 50 ng cDNA of each samples was added up to 25 ml per reaction. qPCR assays were carried out using a StepOnePlus sequence detection system. The cycling conditions were as follows: 10 min of polymerase activation at 95°C, followed by 40 cycles at 95°C for 15 s and 60°C for 60 s. U6 was used as an internal control. For detection of the *HIF-1α* mRNA, PCR amplification consisted of 40 cycles of denaturation at 95°C for 10 s, annealing at 55°C for 10 s and extension at 72°C for 20 s using the following primers: HIF-1A, sense: 5- GAAAGCGCAAGTCTTCAAAG-3, antisense: 5-TGGGTAGGAGATGGAGATGC-3; GAPDH, sense: 5-AATCCCATCACCATCTTCCA-3, antisense: 5-TGGACTCCACGACGTACTCA-3. Experiments were repeated three times using the 2^–ΔΔ*C*^_T_ method.

### miRNAs and plasmid DNA transfection

miRNA mimics (*miR-199a* mimic) and negative control miRNA mimics were purchased from GenePharma (Shanghai, China). Lipofectamine 2000 transfection reagent (Invitrogen) was used for the transfection of miRNAs. *miR-199a* or negative control mimic was transfected at 25 nM. Forty-eight hours after transfection, cells were collected for the downstream experiments. The expression of *miR-199a* was detected by real-time PCR. HIF-1α overexpression vector was transfected using the Lipofectamine 2000 transfection reagent (Invitrogen). Forty-eight hours after transfection, cells were collected for the downstream experiments. The experiments were repeated three times.

### Luciferase assay

The luciferase assay was performed according to a previous report [13], Briefly, cells were seeded in a 24-well plate at a density of 5 × 10^4^/well and cultured overnight. The pGL3-luciferase reporter gene plasmids pGL3-HIF-1A-3′-UTR (WT), pGL3-HIF-1A-3′-UTR (mutant) or the control-luciferase plasmid were cotransfected into the cells with the control mimic or *miR-199a* mimic using Lipofectamine 2000 Reagent (Invitrogen) according to the manufacturer’s protocol. Dual-luciferase activity assays were assayed 48 h after transfection.

### Cell viability assay

Cell viability was evaluated by MTT. Briefly, 1 × 10^4^ cells were plated in 96-well tissue culture plates for overnight. Cells were then exposed to drugs under normoxic or hypoxic conditions. After that, MTT (Sigma, U.S.A.) was added directly into cells and the generated formazan was dissolved in DMSO and the absorbance was recorded. All the experiments were performed in triplicate and repeated three times.

### Western blot

The cellular lysates were prepared by RIPA buffer (Thermofisher Scientific Inc., Waltham, MA). Protein concentration was determined using the Bradford assay (Thermofisher Scientific Inc., Waltham, MA). Proteins were resolved on SDS/PAGE (10% gel) and transferred to immobilon PVDF membranes. Membranes were blocked with 4% BSA for 1 h at room temperature and incubated with the following primary antibodies overnight at 4°C: mouse monoclonal anti-β-actin, (Santa Cruz Biotechnology) or rabbit polyclonal anti-HIF-1α (Santa Cruz Biotechnology). After washing completely in TBS with 0.05% Tween 20 (TBS-Tween), the blots were subsequently incubated with a donkey antirabbit or donkey antimouse peroxidase–conjugated secondary antibody for 1 h at room temperature. The blots were visualized by chemiluminescence methods using the Pierce ECL Western Blotting Substrate (Thermofisher Scientific Inc., U.S.A.).

### Mouse xenograft models

The xenograft experiments were performed as previously described [15]. Osteosarcoma cells were injected subcutaneously into the right flanks of the nude mice. Three weeks after injection, the subcutaneous tumour size had reached a tumour volume of approximately 100 mm^3^. The mice then received intraperitoneal (i.p.) injections of cisplatin (15 mg/kg) twice a week and control mimic or *miR-199a* mimic thereafter.

The present study was carried out in strict accordance with the recommendations in the Animal Care and Use Guidelines of the Department of Orthopedic, First People’s Hospital of Kashgar area (Xinjiang, People’s Republic of China) according to the approved protocols by the Affidavit of Approval of Animal Use Protocol Department of Orthopedic, First People’s Hospital of Kashgar area (Xinjiang, People’s Republic of China). After treatments, mice were killed and tumours were isolated for the downstream analysis.

### Immunohistochemistry

Immunohistochemistry was performed as described previously [16]. The HIF-1α antibody was diluted as 1:200 for immunohistochemical staining.

### Statistics

The values given are mean ± S.E.M. The significance of difference between the experimental groups and controls was assessed by Student’s *t* test. The difference was considered significant with the *P-*value was less than 0.05.

## Results

### *miR-199a* displays tumour suppressor roles in osteosarcoma cells

**Figure 1:**
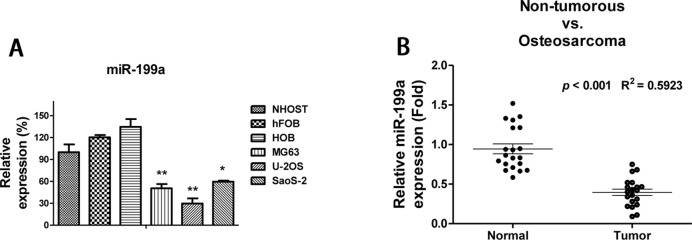
*miR-199a* displays tumour-suppressive functions in osteosarcoma cell lines and patient samples (**A**) Comparison of expressions of *miR-199a* in osteosarcoma cell lines (MG63, U-2OS and SaoS-2) as compared with osteoblast cell lines (NHOST, hFOB and HOB). (**B**) The expressions of *miR-203* were measured by real-time quantitative reverse transcription-PCR (qRT-PCR) in normal adjacent tissues and osteosarcoma tumours. Each group contains 20 patient specimens. Columns, mean of three independent experiments; bars, S.E.M. *, *P*<0.05; **, *P*<0.01; ***, *P*<0.001 and *P*<0.05 is considered to be of statistical significance.

To investigate the potential roles of *miR-199a* in osteosarcoma, we compared the expressions of *miR-199a* in normal osteoblast cells and osteosarcoma cells. As we expected, all three osteosarcoma cell lines showed significantly down-regulated *miR-199a* levels compared with non-tumorous osteoblast cells (Figure 1A), suggesting *miR-199a* might be a tumour suppressor in osteosarcoma. To explore whether *miR-199a* is down-regulated in osteosarcoma tumour tissues, we compared the *miR-199a* expression levels in 20 osteosarcomas and their adjacent normal tissues. Consistently, we observed *miR-199a* levels were significantly down-regulated in 20 cases of osteosarcoma samples compared with normal tissues by TaqMan real-time PCR (Figure 1B). Taken together, our results detected *miR-199a* expressed lower levels in osteosarcoma, suggesting a tumour suppressor role of *miR-199a*.

### Overexpression of *miR-199a* suppresses cell proliferation and motility

**Figure 2:**
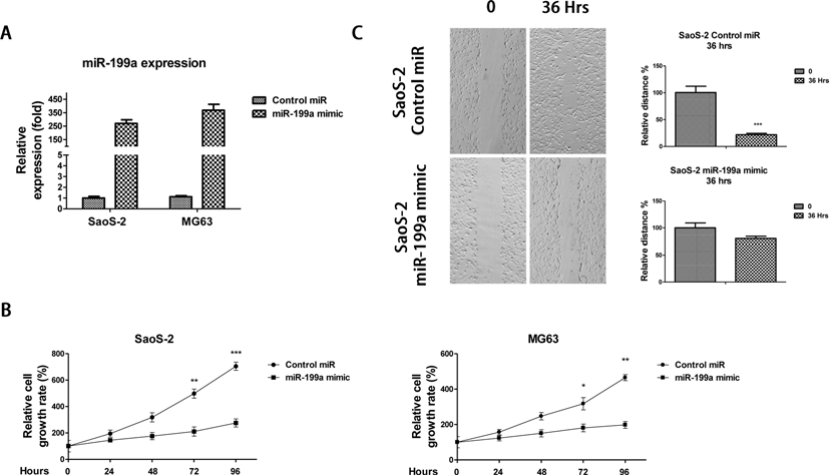
*miR-199a* inhibits osteosarcoma cell migration and proliferation (**A**) SaoS-2 and MG63 cells were transfected with *miR-199a* mimic or scramble control miRNAs for 48 h then the expression of *miR-199a* was analysed by quantitative reverse transcription-PCR (qRT-PCR) and normalized to RNU6. (**B**) SaoS-2 (left) and MG63 (right) cells were transfected with control miRNAs or *miR-199a* mimics for 24 h, MTT assay was performed to measure the cell growth rates every 24 h. (**C**) SaoS-2 cells were transfected with control miRNAs or *miR-199a* mimics for 24 h, then the wound healing assay was performed. *miR-199a* mimics inhibits the migration rates of SaoS-2 cells in 36 h after the scratch. Columns, mean of three independent experiments; bars, S.E.M. *, *P*<0.05; **, *P*<0.01; ***, *P*<0.001 and *P*<0.05 is considered to be of statistical significance.

We next investigated whether exogenous overexpression of *miR-199a* could affect the osteosarcoma cellular processes. To assess the effect of *miR-199a* on the cell growth and motility of osteosarcoma cells, we transfected *miR-199a* mimics into two osteosarcoma cell lines, SaoS-2 and MG63 (Figure 2A). Figure 2B showed in both Saos-2 and MG63 cell lines, cells with *miR-199a* overexpressing demonstrated significantly suppressed proliferation after 72 h compared with cells with control miRNAs transfection. Moreover, we observed a significant decrease in the number of cell migrations in SaoS-2 cells transfected with *miR-199a* after 36-h scratch. (Figure 2C), indicating *miR-199a* might be selected as a target to treat osteosarcoma.

### Cisplatin treatments inhibits *miR-199a* and cisplatin resistant osteosarcoma cells show down-regulated *miR-199a*

**Figure 3:**
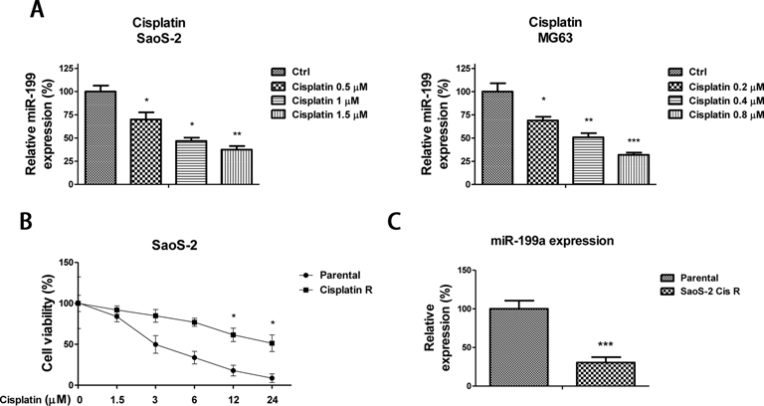
*miR-199a* is negatively correlated with cisplatin sensitivity (**A**) SaoS-2 (left) and MG63 (right) cells were treated with cisplatin at the indicated concentrations for 48 h, then the expressions of *miR-199a* were measured by qRT-PCR. (**B**) SaoS-2 cisplatin resistant and parental cells were treated with cisplatin at 0, 1.5, 3, 6, 12 and 24 μM for 48 h, then the cells’ viabilities were measured by MTT assay. (**C**) The expressions of *miR-203* were measured by qRT-PCR in SaoS-2 cisplatin resistant and parental cells. Columns, mean of three independent experiments; bars, S.E.M. *, *P*<0.05; **, *P*<0.01; ***, *P*<0.001 and *P*<0.05 is considered to be of statistical significance. Abbreviation: qRT-PCR, quantitative reverse transcription-PCR.

As we described above, cisplatin remains the second most commonly used chemotherapy for high-grade osteosarcoma [10,11]. Therefore, we focused on the functions of *miR-199a* in the modulation of cisplatin sensitivity in osteosarcoma. To determine whether *miR-199a* levels are correlated with chemotherapeutic response, we identified the expressions of *miR-199a* before and after cisplatin-based chemotherapy. We found that *miR-199a* expression levels were significantly decreased in SaoS-2 or MG63 cells after gradient cisplatin treatments (Figure 3A), suggesting that *miR-199a* involves the modulation of cisplatin sensitivity in osteosarcoma cells.

To assess the directly regulatory effects of *miR-199a* on cisplatin sensitivity in osteosarcoma cells, we established cisplatin resistant cell line from the SaoS-2 parental cells by treatment of cells with gradually increasing concentrations of cisplatin. The survival cells were collected and pooled for the following experiments. The sensitivities of SaoS-2 parental and cisplatin resistant cell lines in response to increased doses of cisplatin were demonstrated, where cisplatin significantly inhibited proliferation of parental cells over 72 h, whereas the SaoS-2 cisplatin resistant cells were less sensitive to cisplatin at 3–24 μM (Figure 3B). Cisplatin concentration of cisplatin resistant cells (IC 50: 31.51μM) is approximately 10-fold higher than that of the SaoS-2 parental cells (IC_50_: 3.05 μM). As we expected, the expression of *miR-199a* was down-regulated in SaoS-2 cisplatin resistant cells, (Figure 3C). These data indicate that inhibition of *miR-199a* may be applied to the cisplatin-based chemotherapy.

### HIF-1α is correlated with the *miR-199a*-molulated cisplatin sensitivity

**Figure 4:**
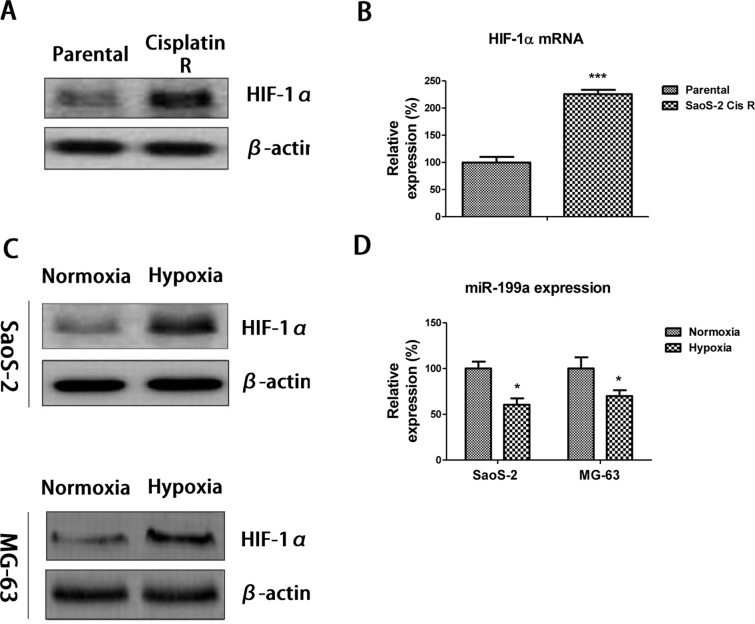
HIF-1α is involved in the *miR-199a*-modulated cisplatin resistance (**A**) The expressions of HIF-1α were measured by Western blot and (**B**) qRT-PCR in SaoS-2 parental and cisplatin cells. β-actin is a loading control. (**C**) The expressions of HIF-1α were measured in SaoS-2 (upper) and MG63 (lower) cells under normoxia or hypoxia by Western blot. (**D**) The expressions of *miR-199a* were measured in SaoS-2 and MG63 cells under normoxia or hypoxia by qRT-PCR. Columns, mean of three independent experiments; bars, S.E.M. *, *P*<0.05; ***, *P*<0.001 and *P*<0.05 is considered to be of statistical significance. Abbreviation: qRT-PCR, quantitative reverse transcription-PCR.

Since, a previous study demonstrated that HIF-1α is the master regulator during hypoxia response, which leads to cancer cells’ chemoresistance via provoking multiple adaptive responses [17]. To investigate the underlying mechanisms for the *miR-199a*-modulated cisplatin sensitivity in osteosarcoma cells, we compared the expressions of HIF-1α in SaoS-2 parental and cisplatin resistant cells. It was interesting to find that HIF-1α is significantly up-regulated in cisplatin resistant cells at both protein (Figure 4A) and mRNA (Figure 4B) levels, intriguing us to explore the correlation of HIF-1α and *miR-199a* in the regulation of cisplatin sensitivities of osteosarcoma cells. As we expected, HIF-1α was induced under hypoxia in SoaS-2 and MG-63 cells (Figure 4C). In contrast, *miR-199a* was suppressed under hypoxia (Figure 4D). These data illustrated the *miR-199a*-modulated cisplatin sensitivity is correlated with HIF1-α expression.

### HIF-1α is a direct target of *miR-199a* in osteosarcoma cells

**Figure 5:**
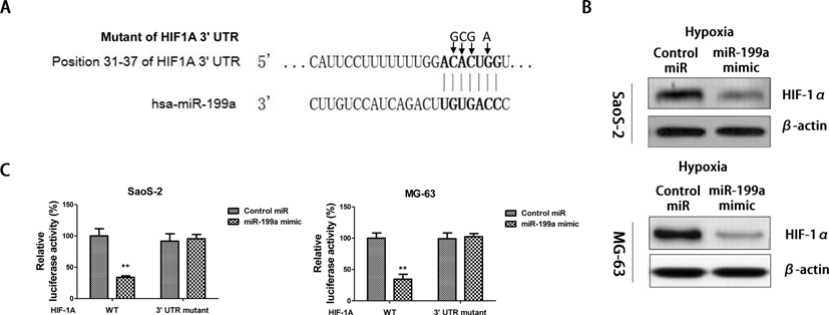
*miR-199a* direct targets 3′-UTR region of HIF-1α (**A**) Target prediction from www.targetscan.org shows the position 31–37 of HIF-1A 3′-UTR contains putative binding sites for *miR-199a*. (**B**) Under hypoxia, SaoS-2 (upper) and MG63 (lower) cells were transfected with control miRNAs or *miR-199a* mimics for 48 h. Cell lysates were prepared for Western blotting analysis. β-actin is a loading control. (**C**) SaoS-2 (left) and MG63 (right) cells were cotransfected with luciferase reporter plasmids with wild-type 3′-UTR of HIF-1A or mutant 3′-UTR of HIF-1A and *miR-199a* mimics or control miRNAs using Lipofectamine 2000 reagent. Forty-eight hours post transfection, cells were harvested and lysed with passive lysis buffer. Luciferase activities were measured by a dual luciferase reporter assay. The results were expressed as relative luciferase activity (firefly LUC/*Renilla* LUC). Columns, mean of three independent experiments; bars, S.E.M. **, *P*<0.01 and *P*<0.05 is considered to be of statistical significance.

We next investigated whether there is direct regulation between *miR-199a* and HIF-1α in osteosarcoma cells. By analysing miRNA target prediction public databases (TargetScan), we noticed that the 3′-UTR mRNA of HIF-1α contains a highly conserved binding site for *miR-199a* (Figure 5A). MG-63 and SaoS-2 cells were transfected with control miRNAs or *miR-199a* mimic. Results showed the expressions of HIF-1α were inhibited by exogenous overexpression of HIF-1α under hypoxia (Figure 5B). To investigate whether *miR-199a* could directly target on 3′-UTR of *HIF-1α* mRNA, sequential replacement of seven base pair region from position 31 to 37 of HIF-1α 3′-UTR was performed to produce the mutant vector. SoaS-2 and MG63 cells were cotransfected with a vector containing pMIR reporter-luciferase fused with original sequence or predicted binding site mutant of the 3′-UTR of *HIF-1α* mRNA and *miR-199a* mimics or control miRNAs. Fluorescence intensity was detected at 24 h post-transfection. The results showed that the luciferase activity of the luciferase reporter gene with the 3′-UTR of *HIF-1α* mRNA was significantly decreased by 66.7%, but that there was no difference in luciferase activity in the cotransfection of vector containing mutant of the 3′-UTR of *HIF-1α* mRNA and *miR-199a* mimics (Figure 5C). These results indicated that HIF-1α expression was significantly inhibited by *miR-199a* through the direct binding to the 3′-UTR region of *HIF-1α* mRNA.

### Overexpression of *miR-199a* sensitizes cisplatin resistant osteosarcoma cells through inhibition of HIF-1α *in vitro* and *in vivo*

**Figure 6:**
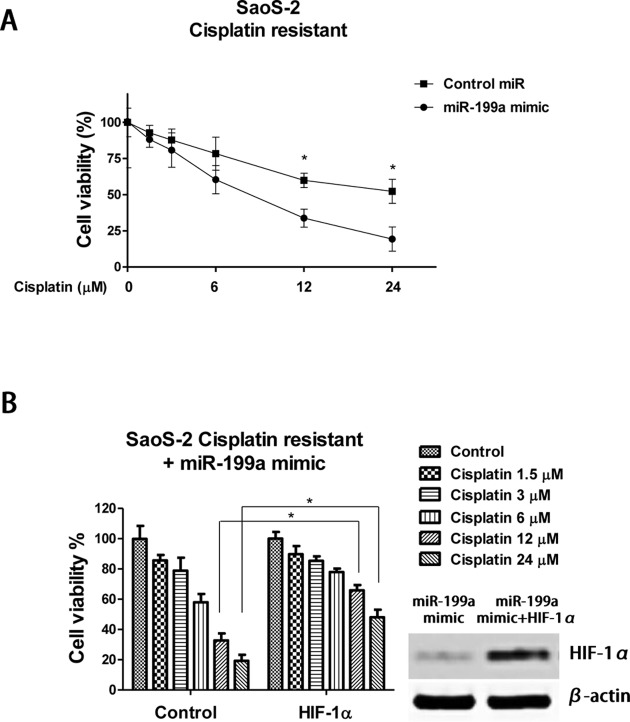
*miR-199a* resensitizes cisplatin resistant osteosarcoma cells through inhibition of HIF-1α (**A**) SaoS-2 cisplatin resistant cells were transfected with control miRNAs or *miR-199a* mimics for 48 h, then the cells were treated with cisplatin at 0, 6, 12 and 24 μM for 48 h followed by cell viability analysis. (**B**) Exogenous overexpression of HIF-1α into *miR-199a* pretransfected SaoS-2 cisplatin resistant cells resulted in tolerance to cisplatin treatments. The expressions of HIF-1α were detected by Western blot. Columns, mean of three independent experiments; bars, S.E.M. *, *P*<0.05 and *P*<0.05 is considered to be of statistical significance.

To study whether targeting miR-199a could resensitize cisplatin resistant osteosarcoma cells to cisplatin, we transfected SaoS-2 cisplatin resistant cells with control miRNAs or *miR-199a* mimics then treated these cells with cisplatin at 0, 6, 12 and 24 μM. Cell viability was assayed to show that cisplatin treatments markedly inhibited viabilities of cells with *miR-199a*-mimics transfection compared with control miRNAs transfection (Figure 6A). Forced expression of *miR-199a* into Saos-2 cisplatin resistant cells decreased the cisplatin IC_50_ from 31.51 to 8.85 μM. To assess whether the *miR-199a* modulated sensitization of cisplatin resistant cells was through the inhibition of HIF-1α, we transfected HIF-1α overexpression vector into *miR-199a* pre-transfected SaoS-2 cisplatin resistant cells to restore the HIF-1α expression to the original level. As we expected, restoration of HIF-1α expression in *miR-199a* overexpressing cells led to a significant tolerance to cisplatin compared with the transfection with control vector in *miR-199a* overexpressing cisplatin resistant cells (Figure 6B), confirming that overexpression of *miR-199a* sensitizes cisplatin resistant osteosarcoma cells to cisplatin by the inhibition of HIF-1α.

**Figure 7:**
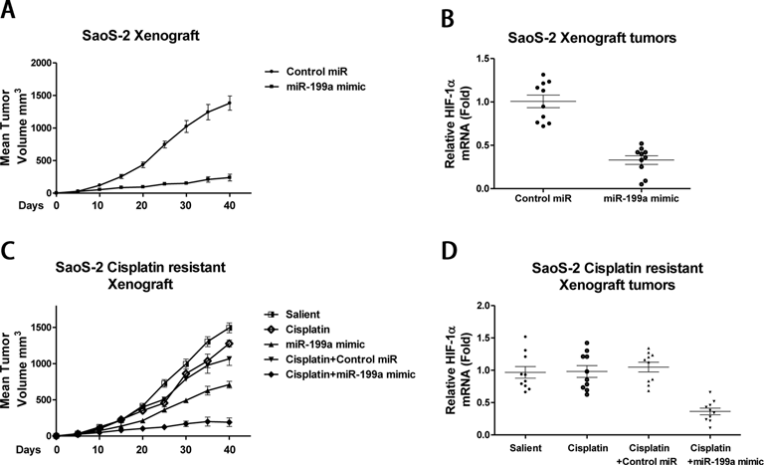
*miR-199a* effectively sensitizes cisplatin resistant osteosarcoma *in vivo* (**A**) The tumour growth from the pre-established SaoS-2 cells with or without transfection of miR-199a mimics derived tumour xenografts. (**B**) The expressions of *HIF-1α* mRNA were measured by qRT-PCR in the pre-established SaoS-2 cells with or without transfection of *miR-199a* mimics derived tumour xenografts. (**C**) The pre-established SaoS-2 cisplatin resistant cells derived xenograft tumours were treated with control (PBS), cisplatin alone (3 mg/kg, two times/week), *miR-199a* mimic alone, cisplatin (3 mg/kg, two times/week) plus control mimic or cisplatin (3 mg/kg, two times/week) plus *miR-199a* mimic. The tumour sizes were measured in 30 days. (**D**) The expressions of *HIF-1α* mRNA measured by qRT-PCR in the Figure 7C described four groups of tumour xenografts. Bars, S.E.M. Abbreviation: qRT-PCR, quantitative reverse transcription-PCR.

Based on the potent anticisplatin resistance activity of *miR-199a* observed in *in vitro* assays, we investigated the roles of *miR-199a* in the regulation of chemosensitivity in an *in vivo* model. The antitumour growth potency was investigated in a subcutaneous mouse model with inoculation of SaoS-2 cells with or without transfection of *miR-199a* mimics into the mammary fat pads of 6-week-old nude mice. Consistent with the *in vitro* results, the *miR-199a* mimic treated mice exhibited a significant reduction in tumour growth (Figure 7A). Moreover, the expression of HIF-1α was down-regulated in *miR-199a* mimics transfected cells derived tumours compared with control miRNAs transfection (Figure 7B). To test whether *miR-199a* could promote the sensitivity of cisplatin resistant osteosarcoma cells, we treated tumour xenografts with cisplatin (3 mg/kg, two times/week) or PBS to mice with injection of SaoS-2 cisplatin resistant cells with or without *miR-199a* transfection once tumours were 100 mm^3^ in size. Our results demonstrated that compared with the control group and *miR-199a* mimic alone group, the combination of *miR-199a* transfection plus cisplatin treatment synergistically inhibited cisplatin resistant cell derived tumour growth (Figure 7C). Remarkably, the expression of HIF-1α was also down-regulated in *miR-199a* mimics transfected cells derived tumours compared with control miRNAs transfection in xenograft tumours (Figure 7D). In summary, our xenograft mouse model revealed *that miR-199a* could sensitize cisplatin resistant osteosarcoma cells through inhibition of HIF-1α *in vivo*.

### Reverse correlation between *miR-199a* and HIF-1α in osteosarcoma patient samples

**Figure 8:**
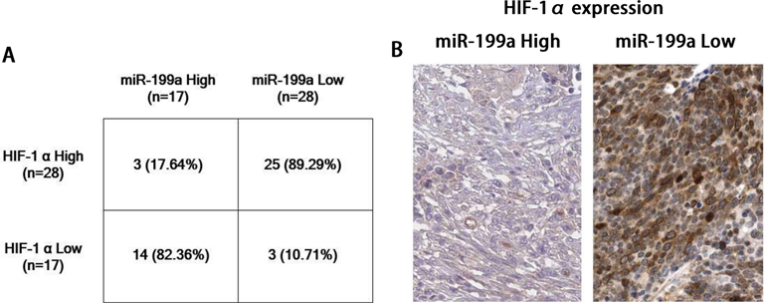
Inverse correlation of expression of *miR-199a* and HIF1-α in human osteosarcoma specimens (**A**) Expression of *HIF-1α* mRNAs were analysed by immunohistochemical staining in *miR-199a* high expressing or low expressing human osteosarcoma tissues. (**B**) Chi-square test analysis of *miR-199a* and *HIF-1α* mRNA expression from osteosarcoma tissues.

The above results demonstrated that HIF-1α is a major target of *miR-199a* in osteosarcoma cell lines and *in vivo* model, we next investigated the correlation between *miR-199a* and HIF-1α expressions in osteosarcoma tissues. We examined *HIF-1α* mRNA expressions in 45 osteosarcoma specimens with immunohistochemical staining. Representative images of HIF-1α expressions in Figure 8A showed that HIF-1α was down-regulated in *miR-199a* high expressed osteosarcoma tissues and highly expressed in *miR-199a* low level osteosarcoma tissues. Statistically, of the 17 osteosarcoma cases with elevated *miR-199a*, 14 (82.36%) of them had low levels of HIF-1α, and 25 of 28 (89.29%) cases with down-regulated *miR-199a* presented high levels of HIF-1α (Figure 8B). These findings illustrated that *miR-199a* regulates HIF-1α expression in clinical osteosarcoma patient specimens.

## Discussion

As we discussed above, *miR-199a* has been reported to be deregulated and negatively correlated with multiple cancer types. In the present study, we found that *miR-199a* levels were significantly decreased in osteosarcoma cancer cell lines and patients. Moreover, forced expression of *miR-199a* suppressed osteosarcoma cell proliferation, consistently with the previous study [18]. Since an increasing number of osteosarcoma patients develop resistance to chemotherapy drugs, we next focused on cisplatin sensitivity of osteosarcoma cells.

miRNAs are found to be novel modulators of cisplatin sensitivity. Galluzzi et al. [19] reported that *miR-181a* could enhance the cisplatin-triggered cell death in A549 cells and *miR-630* could reduce the cisplatin sensitivity in the same cells. However, the precise roles of *miR-199a* in cisplatin resistance in osteosarcoma cells have not been well elucidated. A previous study illustrated that cisplatin treatments decreased *miR-199a* levels in human hepatocellular carcinoma cell lines [13]. In addition, forced expression of *miR-199a* could enhance the cisplatin sensitivity by activating autophagy pathway [13], suggesting that *miR-199a* negatively regulates cisplatin resistance. We demonstrated that cisplatin treatments down-regulated *miR-199a* expressions in osteosarcoma cells. By establishment of cisplatin resistant cell line originating from SaoS-2 cells, we detected that *miR-199a* was down-regulated in osteosarcoma cisplatin resistant cells, intriguing us to explore the mechanisms for the *miR-199a*-modulated cisplatin sensitivity.

HIF-1α is the main transcription factor responsible for the cellular adaptation to hypoxia [20]. Moreover, it has been widely studied that hypoxia commonly exists in solid tumours, leading to cancer cells chemoresistance via activation of adaptive pathways [20]. Other than our above results, some studies have reported that hypoxia can activate autophagy and the hypoxia-induced autophagy may contribute to cisplatin resistance [21]. Our data revealed HIF-1α was up-regulated in cisplatin resistant osteosarcoma cells, indicating that HIF-1α contributes to cisplatin sensitivity. Importantly, we identified HIF-1α as a direct target of *miR-199a* and overexpression of *miR-199a* sensitized cisplatin resistant cells *in vitro* and *in vivo.* In addition, we investigated whether the *miR-199a*-modulated cisplatin sensitization was through the direct inhibition of HIF-1α with the restoration of HIF-1α expression in *miR-199a* overexpressing cells. Here, we show that the rescue of HIF-1α in *miR-199a* overexpressing osteosarcoma cells enhanced resistance to cisplatin, suggesting an *miR-199a*-HIF-1α-cisplatin sensitivity axis in osteosarcoma. Currently, the detailed mechanisms for the HIF-1α-induced cisplatin resistance under hypoxia is still under our investigation.

Taken together, the present study indicates that *miR-199a* contributes to the reversal of cisplatin resistance by blocking the expression of HIF-1α in cisplatin resistant osteosarcoma cancer cells. During this process, HIF-1α is a direct target gene of *miR-199a*. According to the present study, we predict that *miR-199a* may be a potential therapeutic target for cisplatin-resistant osteosarcoma tumours. Our future studies will focus on whether additional molecular mechanisms exist for the HIF-1α-induced cisplatin resistance in an *in vivo* model. This model may be useful for future treatment schedules for overcoming cisplatin resistant osteosarcoma patients.

## Abbreviations

A549: adenocarcinomic human alveolar basal epithelial cells
DMEM: Dulbecco’s Modified Eagle’s Medium
GAPDH: Glyceraldehyde 3-phosphate Dehydrogenase
hFOB: Human Fetal Osteoblast Progenitor Cell Lines
HIF-1α: hypoxia-inducible factor 1α
HIF-1A: hypoxia-inducible factor 1 A
HOB: Human Osteoblasts
MG-63: Human Osteosarcoma MG-63 cell line
NHOST: Normal Human Osteobalsts
pGL3: pGL3-luciferase reporter gene plasmids
pMIR: pMIR-REPORTTM Luciferase
qPCR: quantitative polymerase chain reaction
RIPA: Radioimmunoprecipitation assay
RNU6: RNA, U6 Small Nuclear
SaoS-2: Sarcoma osteogenic cell line
SIRT1: Sirtuin 1
U-20S: Human Bone Osteosarcoma Epithelial Cells
WT: wild type
